# Synthesis and characterization of novel bis(thiosemicarbazone) complexes and investigation of their acetylcholinesterase and glutathione *S*-transferase activities with in silico and in vitro studies

**DOI:** 10.1007/s11030-024-10895-9

**Published:** 2024-06-06

**Authors:** Melike Donmez, Memet Sekerci, Ragip Adiguzel, Ercan Oğuz, Fikret Türkan, Umit Yildiko, Naki Colak

**Affiliations:** 1 Ankara Customs Accounting Directorate, 06590 Ankara, Turkey; 2 Department of Chemistry, Faculty of Science, Firat University, 23119 Elazıg, Turkey; 3 Department of Chemistry and Chemical Process Technologies, Vocational School of Tunceli, Munzur University, 62000 Tunceli, Turkey; 4 Department of Medical Services and Techniques, Health Services Vocational School, Igdır University, 76000 Igdır, Turkey; 5 Department of Basic Sciences, Faculty of Dentistry, Igdır University, 76000 Igdır, Turkey; 6 Department of Bioengineering, Architecture and Engineering Faculty, Kafkas University, 36100 Kars, Turkey; 7 Department of Chemistry, Faculty of Arts and Sciences, Hitit University, 19100 Corum, Turkey

**Keywords:** Bis(thiosemicarbazone) ligands, Metal complexes, Enzyme inhibition, Molecular docking

## Abstract

In this study, firstly, bis(thiosemicarbazone) ligand [L: 2,2′-(2-(2-(4-methoxyphenyl)hydrazineylidene)cyclohexane-1,3-diylidene)bis(hydrazine-1-carbothioamide)] was synthesized by the condensation reaction of thiosemicarbazide and ketone compound (2-(2-(4-methoxyphenyl)hydrazone)cyclohexane-1,3-dione). The metal complexes were synthesized by the reaction of obtained ligand (L) with CuCl_2_·2H_2_O, NiCl_2_·6H_2_O, CoCl_2_·6H_2_O, and MnCl_2_·4H_2_O salts. The structures of synthesized ligand and their complexes were characterized using elemental analysis, IR, UV–Vis, ^1^H-NMR spectra, ^13^C-NMR spectra, magnetic susceptibility, mass spectra (LC–MS), thermogravimetry analysis-differential thermal analysis (TGA-DTA), and differential scanning calorimetry techniques. According to the results of the analysis, square plane geometry was suggested for Cu and Co complexes. However, the structures of Ni and Mn complexes were in agreement with octahedral geometry. Molecular docking analysis and pharmacological potential of the compound were evaluated to determine the inhibitory potential against acetylcholinesterase (AChE) and Glutathione-S-transferases (GST) enzymes. The compound exhibited strong binding/docking indices of − 5.708 and − 5.928 kcal/mol for the respective receptors. In addition, L–Ni(II) complex was found to be the most effective inhibitor for AChE enzyme with a *K*_i_ value of 0.519. However, with a *K*_i_ value of 1.119, L–Cu(II) complex was also found to be an effective inhibitor for the GST enzyme.

## Introduction

Thiosemicarbazones are obtained through the condensation reaction of aliphatic, aromatic, or heterocyclic aldehydes or ketones with thiosemicarbazide compounds. If the ketone compound is a dione, bis(thiosemicarbazone) is formed as a product [1, 2].

Thiosemicarbazones generally act as bidentate ligands, while bis(thiosemicarbazones) act as tetradentate ligands, coordinating with metal ions over nitrogen and sulfur atoms of azomethine [1, 3]. In other words, thiosemicarbazones are chelating ligands that can form a 5-membered chelate ring over the sulfur atom and the azomethine nitrogen atom with transition metal ions [3, 4]. Thiosemicarbazones have attracted a great deal of attention since they were first synthesized, and this interest continues with increasing momentum today, as they have properties such as variable binding modes, structural diversity, promising biological effects, and selective ion sensing capability [1, 3, 4].

The investigation of anticancer and antitumor activities related to the biological effects of thiosemicarbazone complexes is currently a prominent research area. [5]. With the use of platinum complexes in cancer treatment, transition metal complexes began to be used extensively in cancer treatment [6]. However, when it was determined that platinum complexes, which are widely used in cancer treatment, have serious side effects, researchers turned to find alternatives in this regard [6]. In this way, researchers have obtained positive results when they have synthesized metal complexes of various organic ligands and investigated their anticancer activities both in vitro and in vivo [6]. In this context, many studies have been conducted on mono- and bis(thiosemicarbazone) complexes, and it has been determined that Cu(II) complexes effectively show anticarcinogenic activity. Therefore, studies on Cu(II) bis(thiosemicarbazone) derivatives have been continuing intensively since the beginning of the twenty-first century [6].

Palanimuthu et al. reported that certain bis(thiosemicarbazone) copper complexes exhibit selectivity against cells with low oxygen levels and this is due to the retention of Cu(II) in the cell by being partially reduced to Cu(I) [5].

On the other hand, Heribabu et al. investigated the DNA binding affinity of Ni(II) complexes of N-alkylated isatin-based thiosemicarbazone ligands using spectrophotometric methods. As a result of the investigation of the interaction of calf thymus DNA and bovine serum albumin with Ni(II) complexes by absorption and emission spectral methods, the researchers determined that the complexes degraded the DNA without any external agent, and they showed that the interaction of complexes with DNA and protein is supported by molecular docking studies. Additionally, in the in vitro cytotoxicity study of the complexes, they reported that it has significant activity against human breast (MCF7) and lung (A549) cancer cell lines [7].

Anjum et al., suggested a Cu-induced oxidative stress mechanism because some substituted bis(4,4-dimethyl-3-thiosemicarbazone) Cu(II) complexes show antiproliferative activity to a large extent against tumor cells [8].

Singh et al. compared mononuclear and dinuclear Cu(II) thiosemicarbazone complexes with free thiosemicarbazone ligands and several topoisomerase-II inhibitors, and they revealed that the complexes showed significantly higher growth inhibitory activity against tumor cells, while their IC_50_ values were lower [9]. On the other hand, dinuclear complexes were found to have higher anticancer activity than monometallic ones. Cu(II) thiosemicarbazones are reported as more potent antiproliferative agents with less toxic effects and clinically more effective than transition metal complexes such as Ni and Pt [9].

Babak and Ahn [10] investigated the effects of modulating intracellular Cu balance on tumor progression and susceptibility to treatment modalities. In this context, they tried to determine an appropriate method of using Cu depletion/overload conditions to achieve the best possible patient outcome with minimal toxicity. In addition, the advantages of using Cu complexes such as Cu-(bis)thiosemicarbazones and clinical data as anticancer drug candidates are discussed [10].

King et al. determined cellular uptakes and cytotoxicities of biacetylbis(thiosemicarbazone) (ATS), pyruvaldehyde bis(thiosemicarbazone) or glyoxal bis(thiosemicarbazone) (GTS) Co(III) complexes in cancer cells under hypoxic conditions [11]. It was stated that cellular uptake and cytotoxicity were significantly affected by the equatorial nature of the selected bis(thiosemicarbazone) ligands, and the uptake of PTS complexes was much more effective than the others, and with the results obtained, the structure–activity relationship for the rational design of new Co(III) anticancer agents, as well as the relationship between the anticancer potential and the hypoxia targeting properties of the Co(III)/Co(II) redox couple are discussed [11].

Apart from these, thiosemicarbazone Mn(II) complexes have biological properties such as antibacterial and antioxidant activities and play an extremely important role as homogeneous catalysts for oxidation reactions [12]. Enzymes in general play a role in all biochemical activities [13]. All vital activities take place in the presence of enzymes [14]. The hypothesis that xenobiotics consumed by animals are converted into water-soluble substances and excreted from the body through urine was first proposed by R in 1947.T. It was coined by Williams and explained in “Detoxification mechanisms” [15, 16].

Drugs, additives and chemicals that create environmental pollution cause chemical change in the organism catalysed enzymes by stopping, they often lose their effectiveness. Such metabolic changes are called “detoxification.” The detoxification process involves the removal of toxic substances that are to be removed by increasing their solubility in water as a result of an enzymatic reaction and by excretion [17–20].

Glutathione-*S*-transferases (GSTs; EC. 2.5.1.18) are multifunctional enzymes that facilitate the detoxification of various exogenous and endogenous compounds through conjugation reactions with the thiol group of glutathione (GSH). The formed glutathione conjugate is less toxic, and in soluble form, they are excreted from the body [21, 22].

Acetylcholinesterase enzyme (AChE; EC.3.1.1.8) is a non-specific esterase that hydrolyzes acetylcholine to acetate and choline (ACh) [23]. AChE is found in the membrane of erythrocytes, lungs, spleen, and nerve endings. This enzyme hydrolyzes acetylcholine (neurotransmitter) by releasing it from nerve endings. Thus, the nerve impulse is stopped [24]. BChE is synthesized in the liver and released into the plasma. It is also found in the intestinal mucosa, spleen, pancreas, white matter, and many other tissues, except for erythrocytes. Its physiological role is not exactly known. The two enzymes can be distinguished by their different catalytic activities [25].

In this study, we investigated the enzyme inhibition effects of bis(thiosemicarbazone) ligands and Cu(II), Ni(II), Co(II), and Mn(II) complexes synthesized through the condensation reaction of thiosemicarbazide and a hydrazone compound after their structures are elucidated by elemental analysis, thermogravimetry analysis-differential thermal analysis (TGA-DTA), and spectrometric methods. In addition, it was shown that the AChE and GST enzymes' inhibition property of the ligand was confirmed by molecular docking study.

## Experimental section

### Materials and measurements

All solvents were of analytical AR (reagent grade) and the highest purity available. The CuCl_2_·2H_2_O, NiCl2·6H_2_O, CoCl_2_·6H_2_O, and MnCl_2_·4H_2_O, and starting material for the ligand (L) were provided from Sigma Aldrich company. Thiosemicarbazide (99%, pharmacy grade) was acquired from Sigma Aldrich/Merck. The elemental micro-analyses (CHNS) were carried out by a Leco (CHNS-932) instrument. FT-IR spectra were performed utilizing KBr pellets on a Perkin Elmer (Precisely Spectrum One) in the range of 400–4000 cm^−1^. UV–Vis spectra were taken in DMSO by Shimadzu (UV-1700) Spectrophotometer in the extent of 200–800 nm. Magnetic susceptibility measurements were carried out by Sherwood Scientific Magnetic Susceptibility Balance. ^1^H-NMR and ^13^C-NMR spectra were performed on a DMSO-d_6_ and used as a solvent and Bruker (300 MHz Ultrashield TM) spectrophotometer. The mass spectra were taken with Agilent LC/MSD. The TGA-DTA analyses were performed from ambient temperature to 800 °C under a nitrogen atmosphere with a heating value of 10 °C/min used by Shimadzu (TA-60 WS) thermal analysers. The DSC analysis of the ligand was achieved with the Shimadzu DSC 60 A thermal analyser at the same ambient and heating rate as in the TGA analysis. For inhibition studies, GST enzyme, AChE enzyme, substrates glutathione (GSH), and 1 color 2,4 dinitrobenzene (CDNB) and all other chemicals were purchased from sigma Aldrich company.

### Molecular docking studies

Molecular docking was carried out to investigate the binding mechanism of the ligand to the active binding site on the protein. Maestro version 11.8 was used as the platform in the studies [17–19]. High-resolution (1.55–2.10 Å) crystal structures of AChE (PDB ID: 8DT7) and GST (PDB ID: 7BIB) have been downloaded (see http://www.rcsb.org/pdb). The structures of the ligands were converted to Chem bio 3D SDF file format. The structure of the synthesized compound as ligand was prepared according to previous studies using the Ligprep module [26–28]. During protein preparation, all water molecules in the crystalline structure were cleared. According to previous studies, using the receptor grid generation module, the binding sites of the protein were identified and prepared for glide docking. Docking studies were carried out with the Glide docking module. The best binding energies and binding poses between ligand and protein were calculated. The resulting docking interactions were visualized with Discovery Studio version 4.5 [29].

### Synthesis of bis(thiosemicarbazone) ligand (L)

**Scheme 1:**
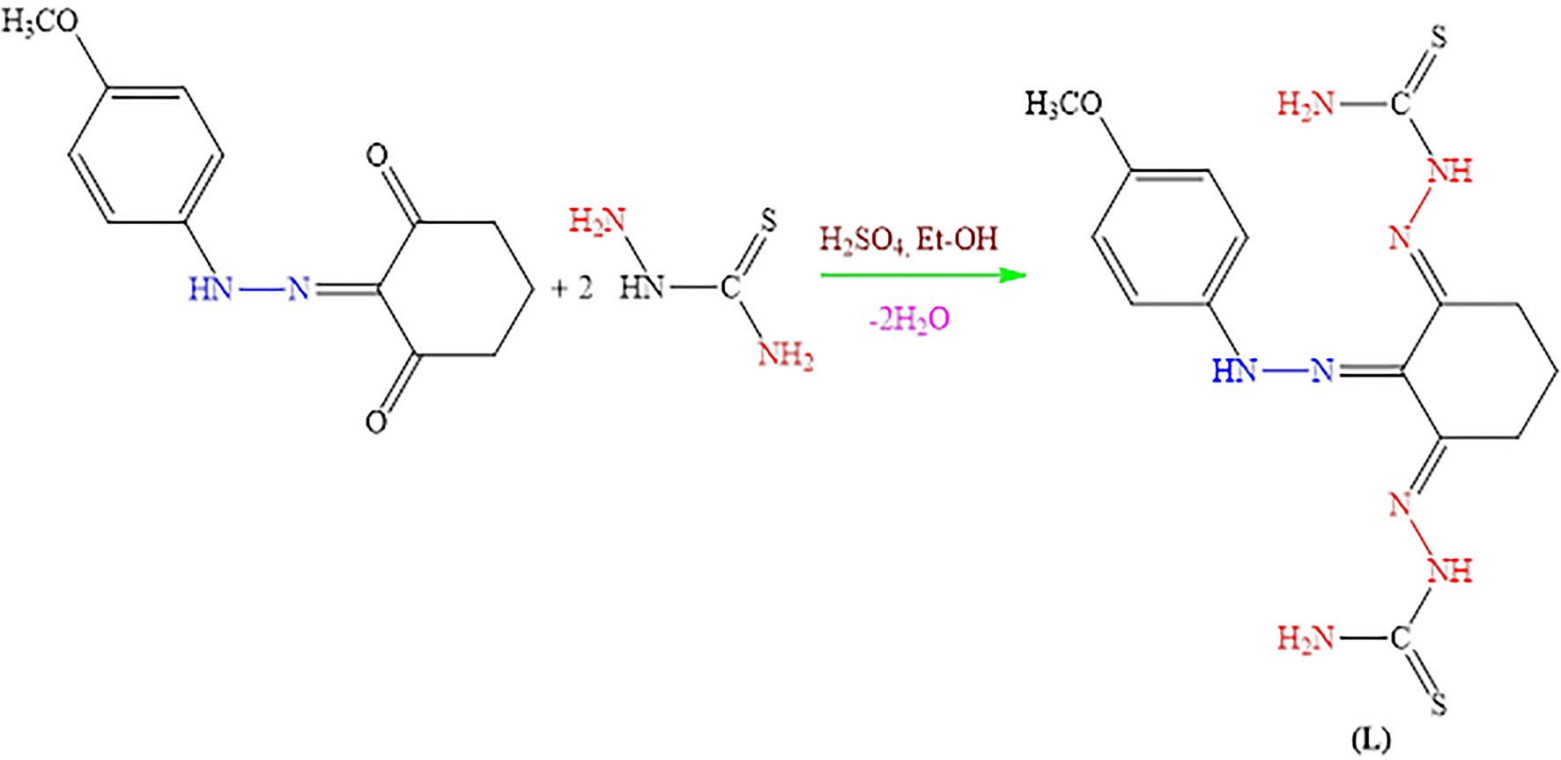
Synthesis reaction of the ligand (L)

The synthesis of bis(thiosemicarbazone) ligand (L) was achieved according to reaction procedure in Scheme 1 [30].

0.91 g (0.01 mol) of thiosemicarbazide was dissolved in 100 mL of hot ethanol that in two neck round bottom glass balloon of 500 mL. 1.23 g (0.005 mol) of 2-(2-(4-methoxyphenyl)hydrazone)cyclohexane-1,3-dione dissolved in 15 mL hot ethanol was added dropwise. After half an hour, three drops of concentrated H_2_SO_4_ were added and then formed a brick-colored precipitate. After refluxing for 2 h, the precipitate was filtered and the brick-colored product obtained by washing several times with hot ethanol was dried in vacuum.

Ligand (**L**): Yield: 96%. Color: Brick color. M.p. (°C): 250. Anal. Calc. for C_15_H_20_N_8_OS_2_: (392,5 g/mol): C, 45.90; H, 5.14; N, 28.54; S, 16.31 Found: C, 46.14; H, 5.65; N, 27.97; S, 16.03. ^1^H-NMR (400 MHz, DMSO-d_6,_ δ ppm): H_11_:12.99,13.37 (1H, s, thiosemicarbazone NH), H_5_: 10.41 (1H, s, hydrazone NH) H_13_: 7,52–8,0, 8,48–8,95 (**2**H, s, NH_2_), H_7_: 7.83 (2H, d, Ar.H) H_8_: 7.04 (2H, d, Ar.H), H_10_: 3.87 (3H, s, OCH_3_), 3.38 (H_2_O residue in DMSO), H_2_: 2.79 (4H, t, CH_2_), 2.51 (proton residue in DMSO) H_2_: 1.79 (2H, t, CH_2_). ^13^C-NMR (100 MHz, DMSO-d_6,_ δ ppm): C_12_: 179.14, 178,58 (***C***=S), C_9_: 151.14, (OCH_3_–***C*** =), C_3_:147.39, 139.84 (***C***=N), C_4_: 131,37 (***C***=N), C_6_: 130,40 (C=***C***–NH), C_7_: 124,26, 121.94 (Ar.***C***), C_8_: 114.26, 111,90 (Ar. ***C***), C_10_: 56.27 (O***C***H_3_), C_2_: 33.68, 27.62 (–***C***H_2_–CH_2_–***C***H_2_–) C_1_: 19.81 (–CH_2_–***C***H_2_–CH_2_–). FT-IR (KBr, cm^−1^) *ν*: 3422, 3224 (NH_2_)_,_ 3165 (N–H, thiosemicarbazone), 3134 (N–H, hydrazone), 2942, 2836 (Al. C–H), 1607 and 1585 (C=N hydrazone and thiosemicarbazone group), 1490, 1276, 1073 and 749 (Thioamide I, II, III and IV bands, respectively, 1019 and 994 (N–N) thiosemicarbazone and hydrazone group. [ESI^+^]: *m/z* 392.5 (Calc.), 393.1 (Found) [M]^+^, 393.5 (Calc.), 394.1 (Found) [M + 1]^+^, 256.5 (Calc.), 257 (Found) base peak of [M–(OCH_3_–C_6_H_4_–NH–N)]^+^.

### Synthesis of the complexes

0.98 g (2.5 mmol) of 2-(2-(4-methoxyphenyl)hydrazone)cyclohexane-1,3-dione bis(thiosemicarbazone) (L) ligand was added to a two neck round bottom glass balloon of 100 mL and it was dissolved in 10 mL hot DMF over a heater magnetic stirrer. A solution of 0.43 g (2.5 mmol) CuCl_2_·2H_2_O salt in 10 mL ethyl alcohol was added dropwise. After a short time, precipitate was observed. Then, the mixture was refluxed for 2 h, the black precipitate formed was filtered. The obtained product was washed several times in ethanol and dried in vacuum.

The same procedure was applied also for 0.60 g (2.5 mmol) NiCl_2_·6H_2_O, CoCl_2_·6H_2_O, and 0.50 g (2.5 mmol) MnCl_2_·6H_2_O salts to synthesise Ni(II), Co(II), and Mn(II) complexes. Unlike the others, during the synthesis of Co and Mn complexes, each mixture was refluxed for 4 h, and the solvent was removed by half.

**[Cu**_**2**_**L**_**2**_**Cl**_**2**_**]∙Cl**_**2**_**∙DMF·3H**_**2**_**O:** Yield: 55%. Color: black. M.p. (°C): 230. (*µ*_eff_): 086 B.M. Anal. Calc. for Cu_2_C_33_H_53_N_17_O_6_S_4_ Cl_4_: (1181 g/mol): C, 33.53; H, 4.49; N, 20.15; S, 10.84 Found: C, 33.06; H, 4.23; N, 19.65; S, 11.01. FT-IR (KBr, cm^−1^) *ν*: 3412, 3267 (NH_2_)_,_ 3159 (N–H, thiosemicarbazone), 2965, 2935 (Al. C-H), 1615, 1600 (C=N thiosemicarbazone group), 1508, 1282, and 751 (Thioamide I, II, and IV bands, respectively), 1047, 1017 (N–N) thiosemicarbazone group, 615 (C–S). UV–Vis in EtOH: (λ max/nm ε/L mol^−1^ cm^−1^): 333, 477–627. [ESI^+^]: *m/z* 800.4 (Calc.), 800.2 (Found) [M–((H_2_N(S)CHNNH_2_)_2_ + 2Cl^−^ + DMF + 3H_2_O)]^+^, 693.3 (Calc.), 693.1 (Found) [M–((H_2_N(S)CHNNH_2_)_2_ + 2Cl^−^ + DMF + 3H_2_O + C_6_H_4_–OCH_3_)]^+^.

**[Ni**_**2**_**L**_**2**_**Cl**_**2**_**(H**_**2**_**O**_**)4**_**]·2H**_**2**_**O:** Yield: 76%. Color: dark brown M.p. (°C): > 360. (*µ*_eff_): 1.34 B.M. Anal. Calc. for Ni_2_C_30_H_50_N_16_O_8_S_4_Cl_2_: (1079.4 g/mol): C, 33.35; H, 4.63; N, 20.75; S, 11.85 Found: C, 32.86; H, 4.32; N, 20.16; S, 11.19. FT-IR (KBr, cm^−1^) *ν*: 3407, 3280 (NH_2_)_,_ 3162 (N–H, thiosemicarbazone), 3056, 2931 (Al. C-H), 1615, 1581, and 1546 (C=N thiosemicarbazone group), 1486, 1280, 1169, and 751 (Thioamide I, II, III, and IV bands, respectively), 1110 (N–N) thiosemicarbazone group, 600 (C–S). UV–Vis in EtOH: (λ max/nm ε/L mol^−1^ cm^−1^): 362, 500, 538. [ESI^+^]: *m/z* 954.2 (Calc.), 953.9 (Found) [M–(H_2_N(S)CHNN)–2H_2_O]^+^.

**[CoLCl**_**2**_**]**^**.**^**0.5**^**.**^**DMF·2.5**^**.**^**H**_**2**_**O:** Yield: 61%. Color: Black. M.p. (°C): 218. (*µ*_eff_): 1.42 B.M. Anal. Calc. for CoC_16.5_H_28.5_N_8.5_O_4_S_2_Cl_2_: (604 g/mol): C, 32.78; H, 4.72; N, 19.70; S, 10.60 Found: C, 32.28; H, 4.06; N, 19.92; S, 10.25. FT-IR (KBr, cm^−1^) *ν*: 3407, 3280 (NH_2_)_,_ 3162 (N–H, thiosemicarbazone), 3056, 2931 (Al. C-H), 1615, 1581, and 1546 (C=N thiosemicarbazone group), 1486, 1280, 1169, and 751 (Thioamide I, II, III, and IV bands, respectively), 1110 (N–N) thiosemicarbazone group, 600 (C–S). UV–Vis in EtOH: (*λ*_max_/nm ε/L mol^−1^ cm^−1^): 345, 491–581. [ESI^+^]: *m/z* 594.9 (Calc.), 595.0 (Found) [M–0.5H_2_O]^+^.

**[Mn**_**2**_**L**_**2**_**Cl**_**4**_** (H**_**2**_**O**_**)2**_**]**·**2H**_**2**_**O:** Yield: 65%. Color: Dark brown. M.p. (°C): 232. (*µ*_eff_): 2.22 B.M. Anal. Calc. for Mn_2_C_30_H_48_N_16_O_6_S_4_Cl_4_: (1109 g/mol): C, 32.46; H, 4.33; N, 20.20; S, 11.54 Found: C, 32.89; H, 4.64; N, 20.80; S, 12.30. FT-IR (KBr, cm^−1^) *ν*: 3425, 3362 (NH_2_)_,_ 3168 (N–H, thiosemicarbazone), 2961, 2836 (Al. C-H), 1597 (C=N, thiosemicarbazone group), 1485, 1283, and 748 (Thioamide I, II and IV bands, respectively), 1078, 1021 (N–N) thiosemicarbazone group, 619 (C–S). UV–Vis in EtOH: (*λ*_max_/nm *ε*/L mol^−1^ cm^−1^): 362, 500, 538. [ESI^+^]: *m/z* 1106.9 (Calc.), 1107.1 (Found) [M–2H]^+^.

### Enzyme inhibition

#### AChE enzyme inhibition study

The in vitro AChE enzyme inhibition potentials of the synthesized complexes were determined according to the Ellman method [31]. 100 µL buffer solution (1 M Tris/HCl; pH: 8.0), 780 µL deionized sample to measure AChE enzyme activities of complex molecules in the reaction in which acetylthiocholine iodide (AChI) and 5,5-dithiobis(2-nitrobenzioc) acid (DTNB) were used as substrate. Different concentrations of the solutions were mixed in test tubes with water, 20 µL of AChE enzyme solution, and 50 µL of DTNB (0.5 mM) and incubated at 20 °C for 15 min. Then, 50 µL of AChI was added. In the last step, the investigated molecules were added sequentially at different concentrations. By applying the Ellman procedure at 412 nm, the absorbents were removed for 5 min.

#### GST enzyme inhibition study

GST enzyme activity measurement Habig et al. [32] was performed at 340 nm for a total of 3 min. It is based on the principle that the product formed by the reaction of 1-chloro 2,4 dinitrobenzene (CDNB) with glutathione (GSH) as a substrate gives maximum absorbance at 340 nm wavelength. Phosphate pH:6.5 was used as buffer solution.

## Result and discussion

In this study, the structure of the newly synthesized (L) ligand was elucidated in light of FT-IR, ^1^H-NMR and ^13^C-NMR spectroscopy as well as UV–Vis and magnetic susceptibility, mass spectra (LC–MS) spectrophotometric methods. DSC analysis of the ligand was also performed. Structural characterization of the newly prepared Cu(II), Ni(II), Co(II), and Mn(II) complexes was performed with the help of FT-IR spectroscopy, UV–Vis and LC–MS spectrophotometry methods, as well as magnetic susceptibility and TGA analysis.

### FT-IR spectra

The absence of C=O peak belonging to the carbonyl group at 1671 cm^−1^ in the FT-IR spectrum of the synthesized bis(thiosemicarbazone) (L) ligand indicates the formation of the compound through the condensation reaction of both C=O groups with thiosemicarbazide, and the C=N stretching vibration band, which emerged at 1585 cm^−1^ belonging to the azomethine group, also supports this [12].

The absence of any band in the range of 2000–2500 cm^−1^ indicates that there is no S–H group in the free ligand and that the compound has tautomer in the form of thion in the solid phase [33–35].

In addition, the observation of the peaks of the characteristic *ν*(C=S) and *ν*(N–H) vibrational bands at 756 and 3164 cm^−1^ also supports the presence of the thion form of the ligand in the solid phase [36, 37]. On the other hand, the stretching vibration *ν*(N–H) observed at 3121 cm^−1^ can be shown as evidence that the hydrazone group in the ligand has an azo-hydrazone tautomer [38, 39].

When the IR spectrum of the complex is examined, it is observed that the C=N vibration of the thiosemicarbazone group azomethine observed at 1585 cm^−1^ in the free ligand shifts to a high frequency in Cu(II), Ni(II), Co(II), and Mn(II) complexes, at 1600, 1615, 1615, and 1597 cm^−1^ is seen, respectively [40]. In addition, the thiosemicarbazone group, which was observed at 1019 cm^−1^ in the IR spectrum of the ligand, appeared in the range of 1047–1110 cm^−1^ in the high wave number of the N–N vibrations in the complexes [12]. These results indicate that the ligand is coordinated through the nitrogen atom of azomethine [12, 40].

On the other hand, the fact that an N–N vibration band is observed again at 1017, 1018, and 1021 cm^−1^, respectively, in Cu(II), Co(II), and Mn(II) complexes, and besides the thioamide bands given in detail in the experimental section, the presence of a broad C–S band at 617, 615, and 619 cm^−1^ , respectively, confirms that the ligand is unilaterally coordinated and that the thiosemicarbazone group is in the thiol form. In addition, the S–H vibrations observed in the IR spectrum of Cu(II), Co(II), and Mn(II) complexes at 2112, 2077, and 2070 cm^−1^, respectively, also support that the coordination is in the thiol form. However, the absence of the S–H band in the Ni(II) complex supports the detachment of protons from the C–SH group of the thiosemicarbazone [35]. Additionally, *ν*(N–H) vibrations at 3159, 3162, 3172, and 3168 cm^−1^ in the spectrum of Cu(II), Ni(II), Co(II), and Mn(II) complexes are thought to belong to thiosemicarbazone groups that do not participate in coordination.

Finally, the disappearance of the hydrazone group N–H, N–N, and C=N peaks observed in the free ligand at 3134, 994, and 1606 cm^−1^, respectively, in all complexes indicates that the hydrazone group is in the diazo form (see “Experimental section” section).

### NMR studies

**Fig. 1:**
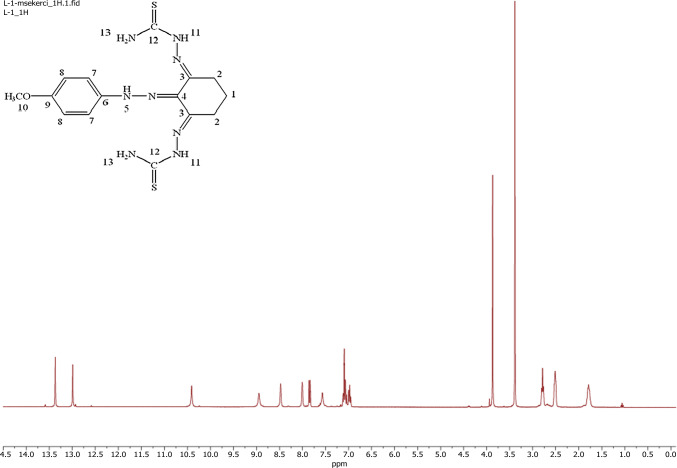
^1^H-NMR spectra of the ligand (L)

In the ^1^H-NMR spectrum of the ligand (see Fig. 1), the N–H proton of the hydrazone group was observed at 10.41 ppm [35, 39], while the peaks of the N–H protons in the thiosemicarbazone group (N–H and -NH_2_) were observed at 12.99 and 13.37 ppm [41]. In addition, the absence of the S–H proton peak around 4.00 ppm confirms that the ligand remains in thion form even in the solvent [42]. In the spectrum of the ligand, the proton signals belonging to the -NH_2_ groups appear as two separate large singlet peaks in the range of 7.52–8.95 ppm, indicating that the free rotation around the C-N bond is blocked due to the partial double bond character [36, 37, 43].

**Fig. 2:**
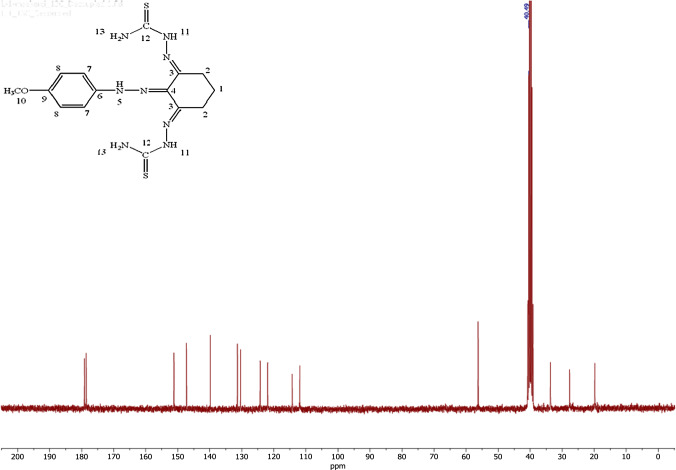
^13^C-NMR spectra of the ligand (L)

In the ^13^C-NMR spectrum of the ligand (see Fig. 2), it is observed that the carbon of the C=S group is in the range of 178.58–179.14 ppm [44], the C=N carbon of the hydrazone group is at 131.37 ppm, and the C=N carbon of the thiosemicarbazone group is between 139.84 and 147.39 ppm. It proves that the recommended structure has occurred. All data belonging to both spectra are given in detail in “Experimental” section.

### Mass spectra

**Fig. 3:**
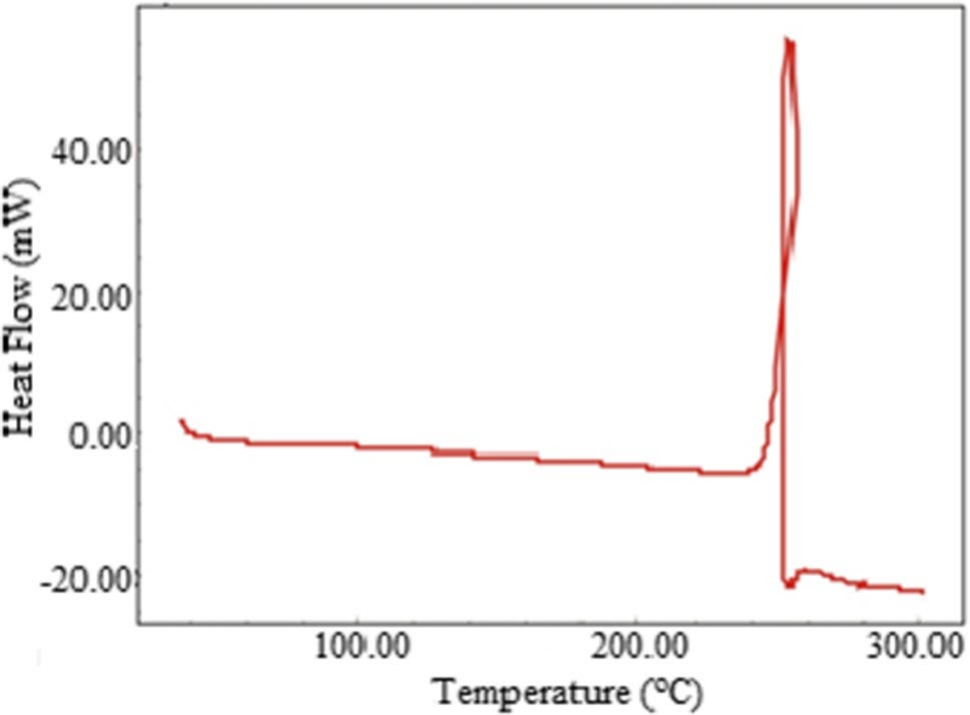
DSC curve of the ligand (L)

The mass spectra of the ligand (L) contain peaks attributable to the given molecular ion, *m/z* 393,1 [M^+^], 394.1 [M + 1]^+^, and base peak m/z 257 [M–(OCH_3_–C_6_H_4_–NH–N)]^+^. In the mass spectra of Cu(II) complex was observed the peak of the cationic complex and the base peak instead of molecular ion peak m/z 800.2 [M-((H_2_N(S)CHNNH_2_)_2_+2Cl^−^+DMF+3H_2_O)]^+^ and 693.1 [M-((H_2_N(S)CHNNH_2_)_2_+2Cl^−^+DMF+3H_2_O+C_6_H_4_-OCH_3_)]^+^, respectively. As for the mass spectra of the Ni(II) complex, was observed as molecular ion peak m/z 953.9 [M-(H_2_NCSNHN+2H_2_O)]^+^. For the Co (II) complex, the theoretical value is in compliance with the experimental result m/z 595.0 [M–0.5H_2_O]^+^. Different from the others, the molecular ion peak in the mass spectra of Mn(II) complex was found m/z 1107.1 [M–2H]^+^. The significances that are used as a base have high abundance in the mass spectra [45, 46] (Fig. 3).

### Electronic spectra and magnetic properties

**Fig. 4:**
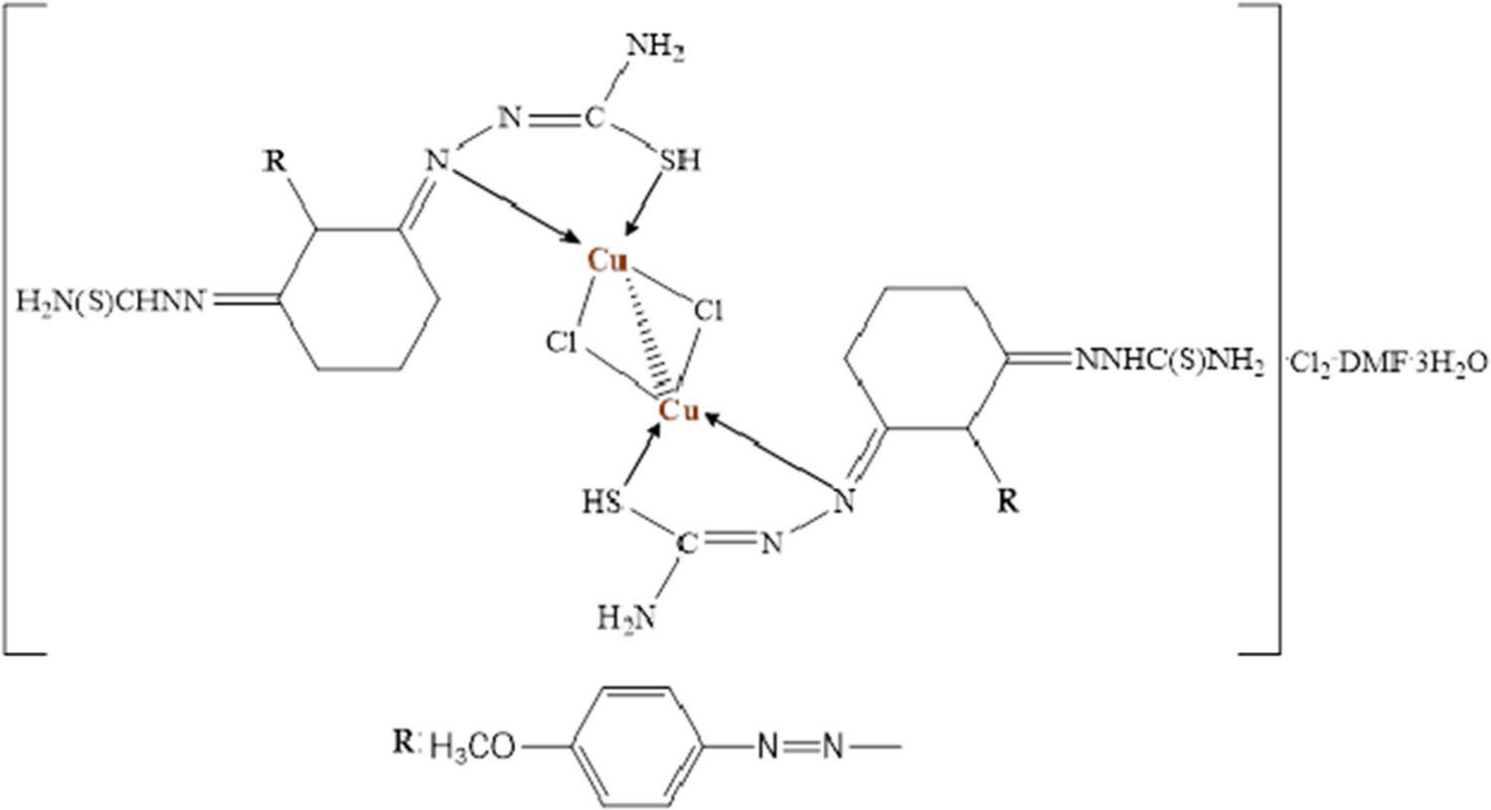
Structure of L–Cu(II) complex

In the UV–visible spectroscopy of Cu(II) complex, the observed broad absorption band in the range of 477–627 nm corresponding to the ^2^B_1g_ → ^2^E_g_ transition indicates the complex has square planer geometry (see Fig. 4). In addition, high-intensity absorption band that observed at 333 nm is regard to charge transfer transition (LMCT) [47].

The *μ*_eff_ value of the binuclear Cu(II) complex was found 0.86 B.M. as to the magnetic susceptibility measurements. The fact that this value is significantly lower than 1.73 B.M corresponding an unpaired electron can be explained by the Cu–Cu interaction due to the Cu–Cl–Cu bridge [48].

The magnetic susceptibility value (*µ*_eff_) for binuclear octahedral Ni(II) complex was determined as 1.34 B.M. The fact that this value is less than 2.83 B.M, corresponding to the two unpaired electrons, indicates the complex including the Ni–Cl–Ni bridge has a strong Ni–Ni interaction [49].

**Fig. 5:**
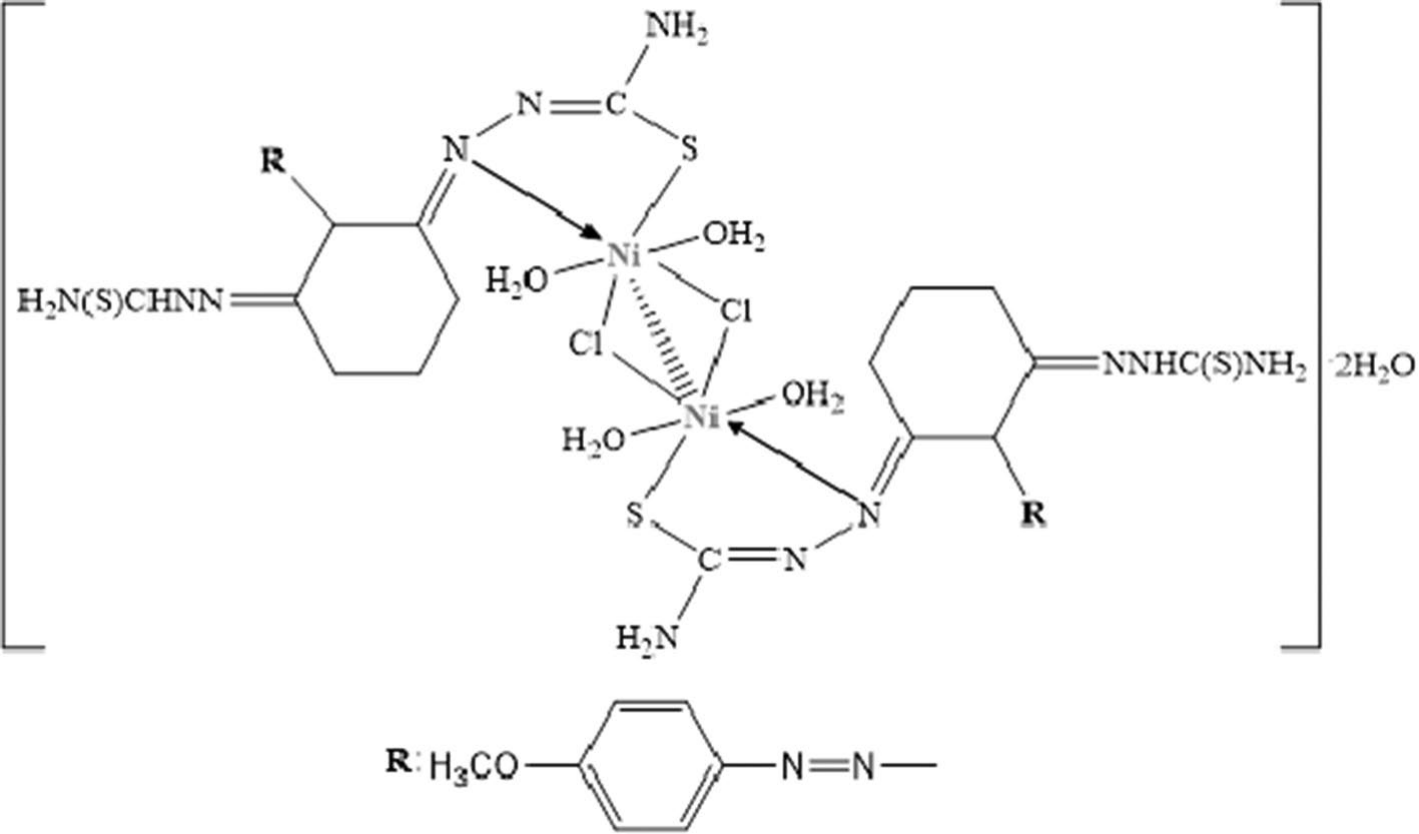
Structure of L–Ni(II) complex

In the UV visible spectra of Ni(II) complex that the observing the transition of ^3^*A*_2g_ (F) →^3^*T*_2g_ (P) (ν^3^) at 500 nm and the transition ^3^*A*_2g_ (F)→^3^*T*_1g_ (F) (ν_2_) at 538 nm indicates the Ni(II) complex has an octahedral geometry (see Fig. 5) [49]. Additionally, the absorption band that observed at 362 nm is corresponded to the charge transition LMCT.

**Fig. 6:**
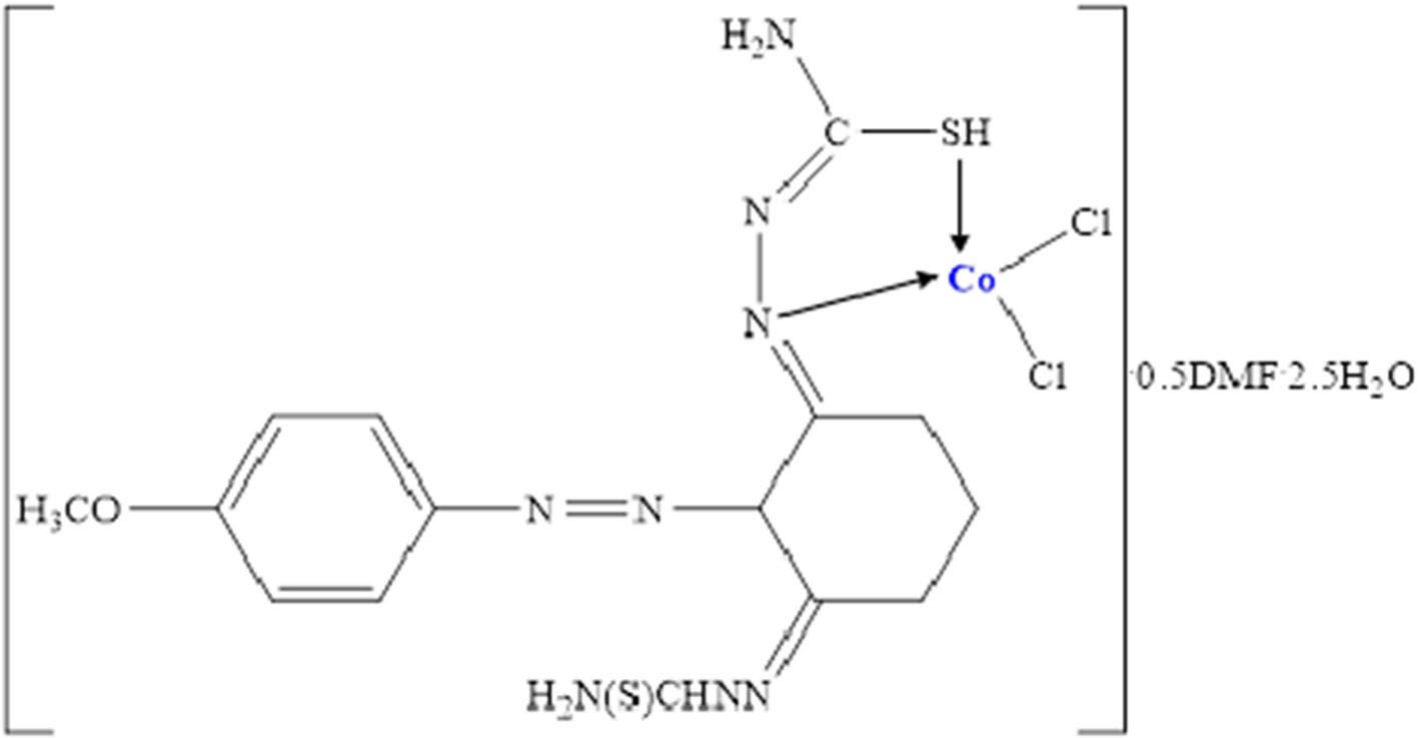
Structure of L–Co(II) complex

As for the Co(II) complex, the *µ*_eff_ value measured as 1.42 B.M indicates that the complex geometry can be square planer or low spin octahedral geometry. However, in the UV–Vis spectra of the Co(II) complex, the band that observing in the range of 491–581 nm and corresponding the ^2^*A*_1g_ → ^2^*E*_g_ transition confirms the complex has square plane geometry (see Fig. 6) [50, 51].

Magnetic susceptibility of the Mn(II) complex was measured as 2.22 B.M. It is reported in the literature that the magnetic moment values of the low spin Mn(II) complexes are in the range of 1.7–2.3 B.M. Based on these results, a low spin octahedral structure is proposed for the Mn(II) complex [52].

**Fig. 7:**
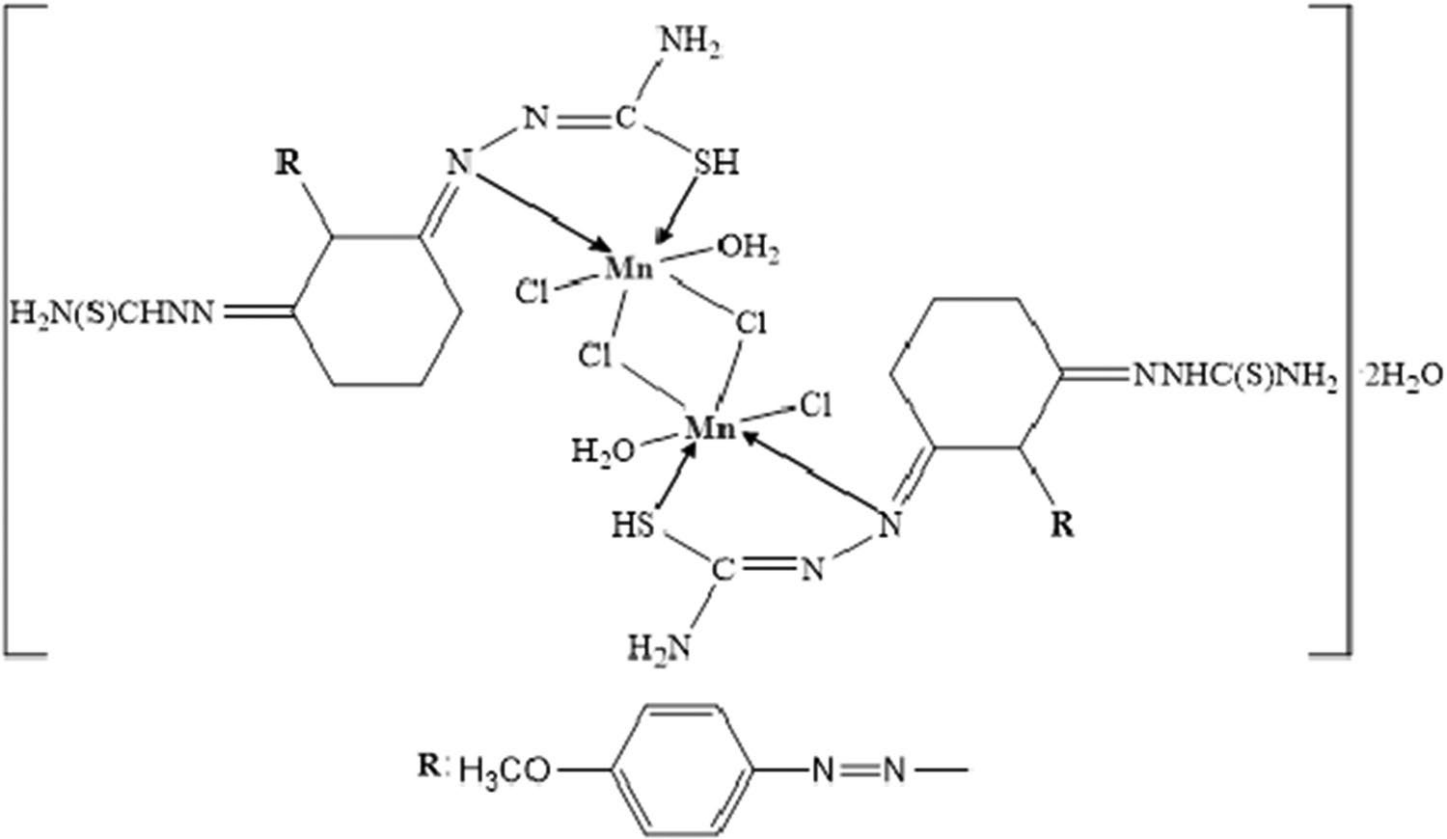
Structure of L–Mn(II) complex

On the other hand, broad shoulders observed in the range of 365–411 nm, 432–504 nm, and 541–585 nm in the UV–Vis spectra indicate that the structure is in low spin octahedral geometry (see Fig. 7) [52, 53].

### TGA and DSC analyses

As a result of the examining the TGA curves of the complexes, it was determined that the Mn(II), Ni(II), Cu(II), and Co(II) complexes degraded in three, four, five, and six steps, respectively. TGA curves were interpreted by considering the mass losses of Cl^−^ ions coordinated to the lattice water, coordinate water, counterion, and metal ion contained in the complexes, as well as the organic parts in general. Mass losses of lattice water contained in the complexes were in the range of 30–125 °C [54] [55]. In the Cu(II) complex, two moles of Cl^−^ counter ions were separated with one mole of DMF in the range of 125–268 °C [55], while two mole of bridge Cl^−^ ions was separated from the complex in the range of 268–315 °C [54]. It was observed that from the TGA curve of the Ni(II) complex, 2 mol of bridge Cl^−^ ion together with four moles of coordinate water was separated from the complex compound up to 250 °C [54–56]. From the TGA data of the four-coordinated Co(II) complex, it was observed that 2.5 mol of crystal water was removed from the complex up to 118 °C and 0.5 mol of DMF up to 240 °C. In addition, it was observed that 2 mol of Cl^−^ ion coordinated to the metal ion [56], together with the C_6_H_4_-OCH_3_ group, was separated from the structure of the complex in the range of 240–444 °C. In the Mn(II) complex, it was observed that 2 mol of crystal water up to 108 °C, along with 2 mol of coordinate water in the range of 108–267 °C, moved away from 2 mol of coordinate Cl^−^ and bridge Cl^−^ ions in the coordination sphere [55, 56]. Finally, the final residue is CuS for the Cu(II) complex and MnO for the Mn(II) complex. In contrast, the final residual product for Ni(II) and Co(II) complexes is metallic Ni and Co. Apart from these, the observation of a single peak in the DSC curve of the (L) ligand at 250 °C indicates the purity of the ligand obtained (see Fig. 3). See Table 1 for all mass losses and more detailed information.

**Table 1:** TGA analysis results of the complexes

Compounds	Mass loss%, found (calc.) decomposition group					
Molecular weight (g/mol)	1st step (°C)	2nd step (°C)	3rd step (°C)	4th step (°C)	5th step (°C)	Residue
L–Cu(II)	52–125	125–268	268–315	315–358	358–625	2CuS
1181	4.56 (4.57)	13.16 (12.19)	6.08 (6.01)	4.80 (5,25)	55.11 (55.80)	16.29
	3H_2_O lw	DMF, 2Cl^−^ ci	2Cl^−^ b	2-OCH_3_	rop	− 16.18
L–Nı(II)	30–70	70–250	250–430	430–650		2Ni
1079.4	3.73 (3.34)	13.71 (13.25)	36.75 (36.36)	34.98 (36.17)		10.83
	2H_2_O lw	4H_2_O cw,	C_6_H_4_–OCH_3_,	NN(C_6_H_7_) NNCSNH_2_ groups		− 10.88
		2Cl^−^ b	ftsc groups			
L–Co(II)^a^	30–118	118–240	240–444	444–495	495–564	Co
604	8.01 (7.46)	7.04 (6.06)	30.54 (29.48)	5.57 (4.64)	38.11 (37.14)	10.73
	2.5H_2_O lw	0.5 DMF	2Cl^−^,C_6_H_4_–OCH_3_	N=N	H_2_NSCHNN(C_6_H_7_) NNCNH_2_	− 9.76
L–Mn(II)	30–108	108–267	267–700			2MnO
1109	3,31 (3,24)	15.39 (16.03) 2H_2_O, cw,	68,87 (67.94)			12.43
	2H_2_O lw	2Cl^−^ b, 2Cl^−^ cs	rop			− 12.79

*b* bridge, *ci* counterion, *cs* coordination sphere, *cw* coordinated water, *ftsc* free thiosemicarbazone, *lw* lattice water, *rop* remaining organic part

^a^6th step: 564–597 °C 4,47 (5,46), SH group

### Enzymes inhibition results

**Fig. 8:**
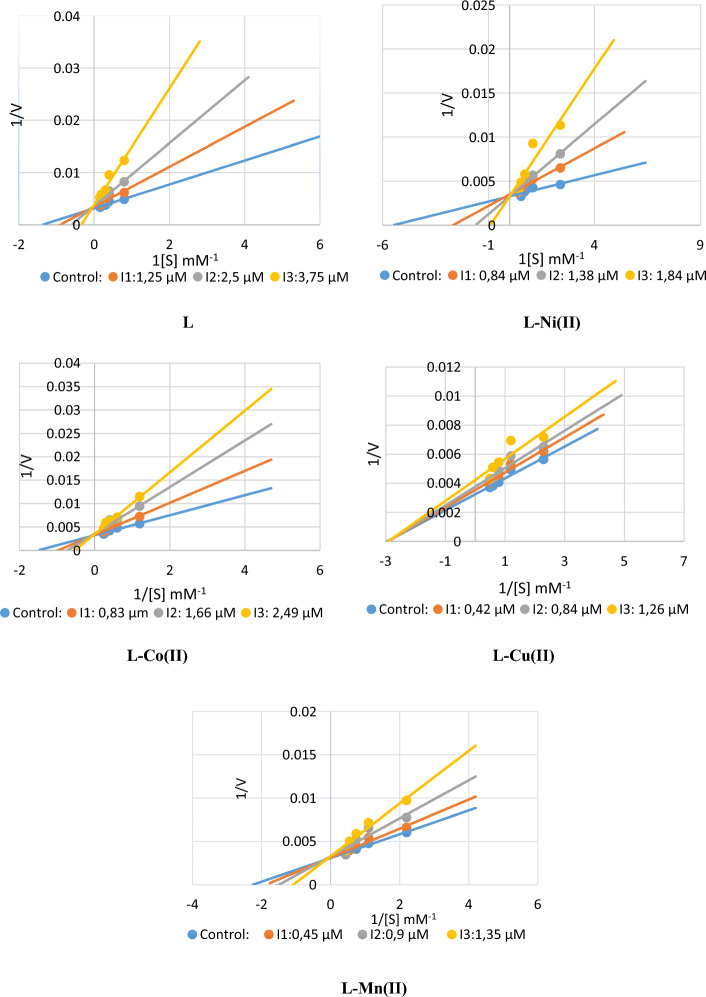
Lineweaver–Burk graphs for the AChE enzyme of complex molecules

All the complex molecules used in this research paper were found to act as inhibitors of both GST and AChE enzymes. In the study, molecules for the AChE enzyme were compared with the standard inhibitor tacrine, and ethacrynic acid was used as standard for the GST enzyme. Inhibition for both enzymes was carried out in two steps. First, the IC_50_ values of the molecules were determined. Then, a K_i_ study was performed using three of these values. AChE enzyme inhibition findings are given below:

IC_50_ values were found in the range of 0.883–2.74. While L–Cu(II) complex had the lowest IC_50_ value, Ligand showed the highest IC_50_ value with 2.74. *K*_i_ values was in the range of 0.569 ± 0.2231–3.839 ± 1.0245. When all the results for the AChE enzyme are evaluated, it is seen that L–Ni(II) complex molecule is the best inhibitor. The tacrine molecule was studied as a standard inhibitor, and the IC_50_ value was 0.848 and the *K*_i_ value was 4.556 ± 0.869. It was discovered that every complex’s output exceeded the norm. Figure 8 depicts Lineweaver–Burk graphs and computed *K*_i_ values and inhibition types. These molecules can be considered superior inhibitors compared to the tacrine molecule. Finding inhibition at the micromolar level is crucial for the development of novel drugs. Obtaining data at this level in our study are critical to ensuring that alternatives to the typical inhibitor tacrine are introduced to the market. Similar study results are available in the literature and appear to be consistent with our findings [17–19, 57].

**Fig. 9:**
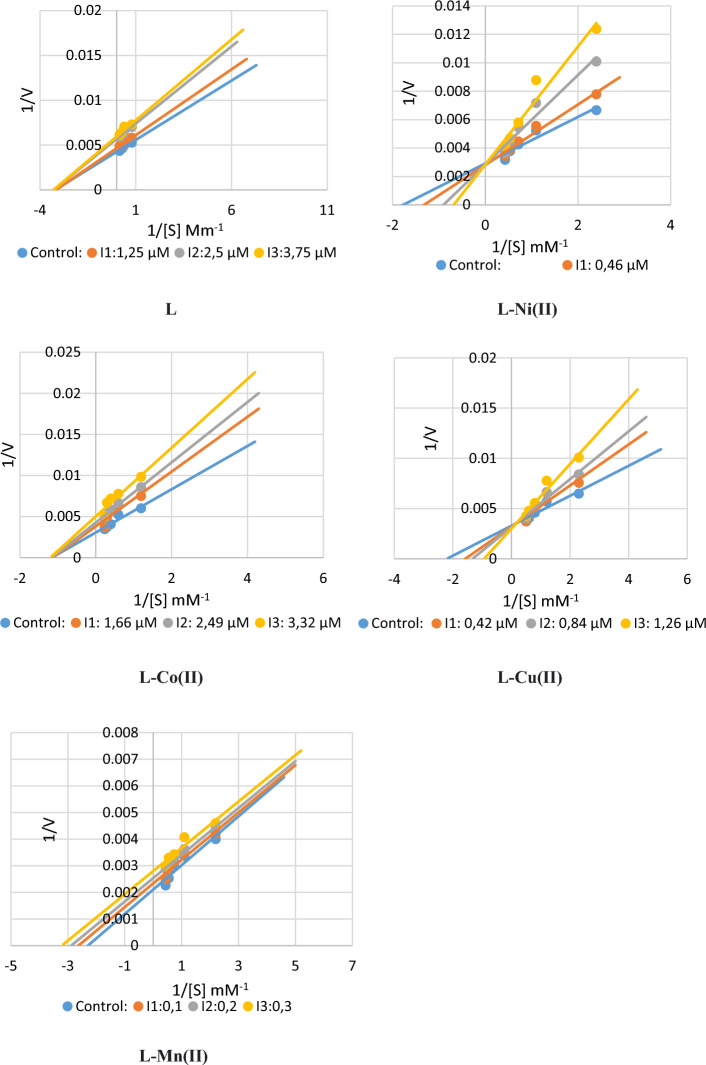
Lineweaver–Burk graphs for the GST enzyme of complex molecules

When the GST enzyme inhibition results are examined, it is seen that the IC_50_ values are in the range of 0.874–2.987. Among these molecules, the L–Mn(II) complex appears to have a lower IC_50_ value. Similarly, when the *K*_i_ values are examined, it is seen that these values are 1.119 ± 0.1796–11,677 ± 5.4471. When all the inhibition results are examined, it is seen that the most effective molecule inhibiting the GST enzyme is L–Cu(II) complex molecule. Ethacrynic acid (EA) is a well-known GST enzyme gold inhibitor. All other molecules—aside from ligand—are superior inhibitors than EA when the study’s results are compared to the standard. The prevalent disease of our time, cancer, is treated therapeutically with medications that inhibit the GST enzyme. Our study is crucial since it has identified novel medications that provide an alternative to EA, the most used standard. As seen in Fig. 9, Lineweaver–Burk graphs were created, and *K*_i_ values and inhibition types were computed. Similar studies are available in the literature and are consistent with our study findings [58, 59]. All the inhibition research findings are given in Table 2.

**Table 2:** Glutathione *S*-transferase, acetylcholinesterase, inhibition effects of the ligand (L), and their complexes

Compounds	IC_50_ (µM)				Ki (µM)	
	AChE	R^2^	GST	R^2^	AChE	GST
L	2.74	0.9957	2.193	0.987	1.2577 ± 0.0724	11.677 ± 5.4471
L–Ni(II)	1.063	0.935	1.063	0.9773	0.569 ± 0.2231	1.4017 ± 0.6088
L–Co(II)	2.07	0.9818	2.987	0.9458	1.422 ± 0.0832	6.402 ± 0.9699
L–Cu(II)	0.883	0.9419	1.17	0.9511	3.839 ± 1.0245	1.119 ± 0.1796
L–Mn(II)	0.90	0.9854	0.874	0.9592	1.781 ± 0.04022	7.0503 ± 2.0138
*Tacrine	0.848	0.9962	–	–	4.556 ± 0.869	–
*Ethacrynic acid	–	–	0.374	0.9632		9.386 ± 3.298

*Standard inhibitors for the ACHE and GST enzymes

### Molecular docking

**Fig. 10:**
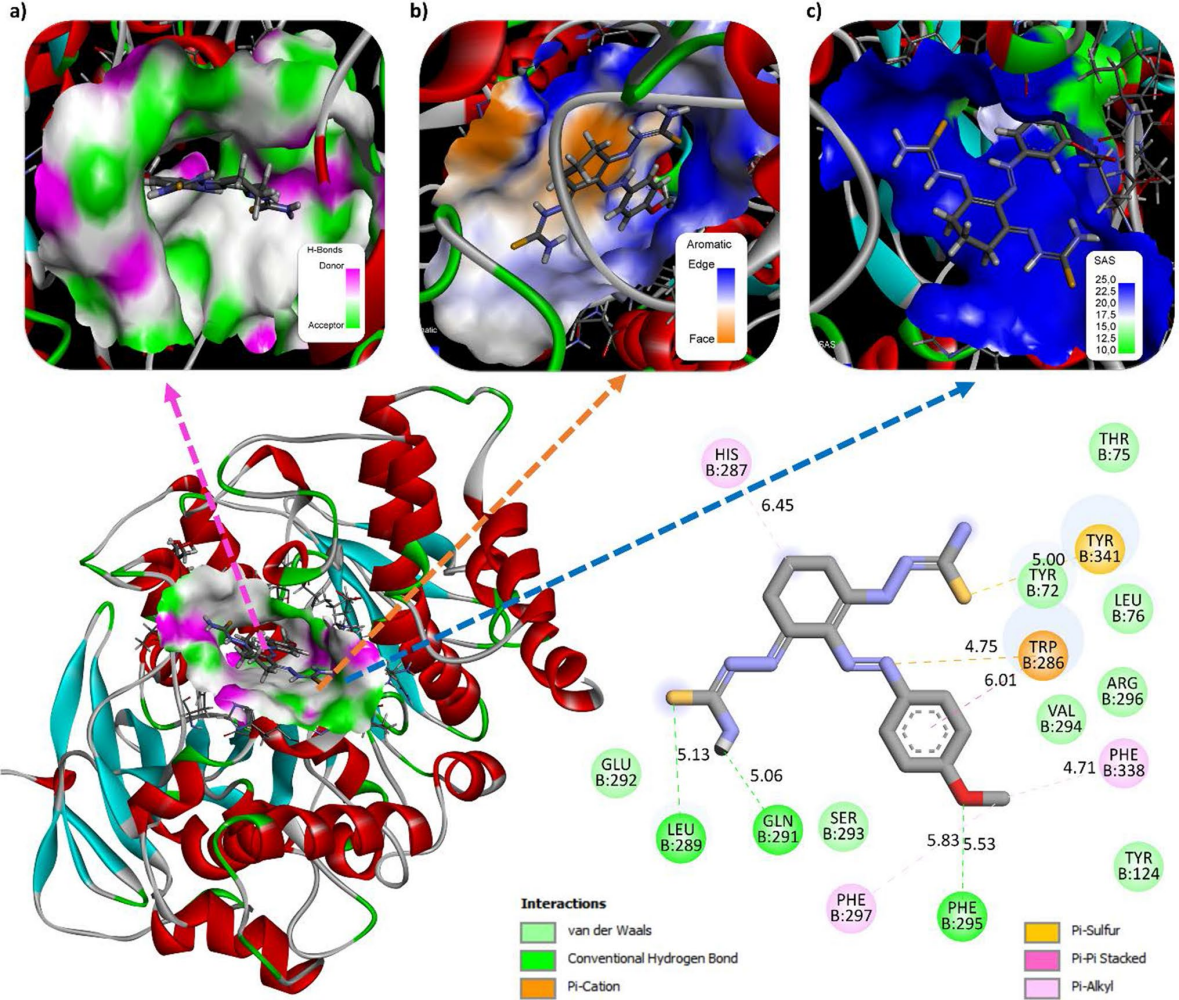
Mode of interaction with ligand–protein enzymes; 3D view of **a** hydrogen bond donor/acceptor surface, **b** aromatic surface and **c** solvent interactions

The molecular docking method is used to investigate the biochemical performance of compounds, categorize protein-ligand interaction and drug design studies. This study is the first and most useful step in the production of new drugs. In this study, the in vitro enzyme inhibition activity of the synthesized ligands was experimentally confirmed. Acetylcholinesterase (acetylcholinesterase) inhibitors are often used to improve symptoms of dementia caused by Alzheimer’s disease [45, 60, 61]. To confirm the inhibition effect mode of the synthesized compound at the molecular level, the interactions of the enzyme with the protein receptor molecules were analyzed by molecular docking and the results are shown in Fig. 10. Acetylcholinesterase (acetylcholinesterase) (AChE) inhibitors are often used to improve symptoms of dementia caused by Alzheimer’s disease. In this interaction, the binding energy of − 5.708 kcal/mol was calculated, showing effective binding to the AChE protein structure. The molecules SER 293, GLU 292, TYR 72, LEU 289, and 5.13 Å are examples of van der Waals bonds to the acetylcholine esterase active site. A stronger hydrogen bond is established between the oxygen and nitrogen atoms in the ligand and the hydrogen atom in the chosen AChE protein, relative to the major contribution of the protein–ligand interaction. For instance, it was discovered that PHE 295 5.53 Å and GLN 291 5.06 Å could establish strong conventional hydrogen bonds. PHE 338 4.71 Å, PHE 297 *π*-alkyl, TYR 341 5.00 Å, *π*-sulfur, TRP 286 6.01 Å *π–π* stacked, and TRP 286 4.75 Å *π*-cation are also present. This highly active compound is also thought to be capable of strong enzyme inhibition due to its ability to make hydrogen bonds [62]. According to the results of the molecular docking analyses obtained in this study, the compound binds to the structure by interacting with the active sites of the protein and shows that it is biologically active.

**Fig. 11:**
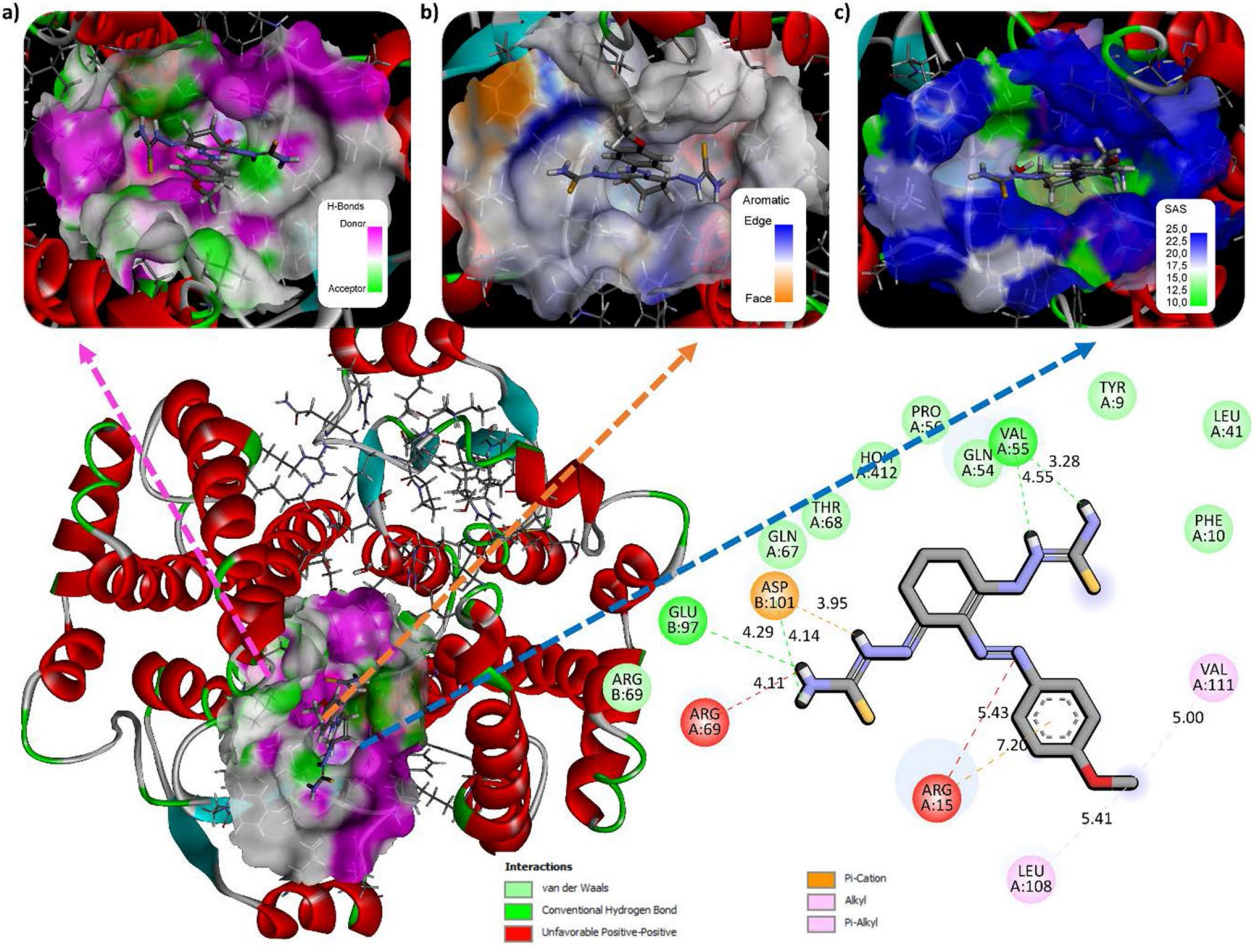
Mode of interaction with ligand–protein enzymes; 3D view of **a** hydrogen bond donor/acceptor surface, **b** aromatic surface and **c** solvent interactions

The compound is bound to the catalytic active site of the enzyme with a total binding energy of − 5.928 kcal/mol by intermolecular interaction. This docking was analyzed based on the best mode and selected as the pose in Fig. 11. The compound showed good binding affinity toward the AChE enzyme based on the scores. Amino acid sequences in the protein structure ARG 69, GLN 67, THR 68 van der Waals interaction with the aromatic ring of the compound, GLU 97 4.29 Å ASP 101 4.14 Å VAL 55 4.55 Å and 3.28 Å show hydrogen bond with functional groups, LEU 108 5.41 Å VAL 111 5.00 Å alkyl interaction with (C=S) groups can be given as examples. Molecular docking studies also supported the inhibitory activities of the compounds and helped to understand the various interactions between ligands and enzyme active sites [63, 64]. The effectiveness of this compound in the design of new potential inhibitory drugs and its mechanism of action has been investigated by docking study.

### Pharmacokinetic analysis

**Fig. 12:**
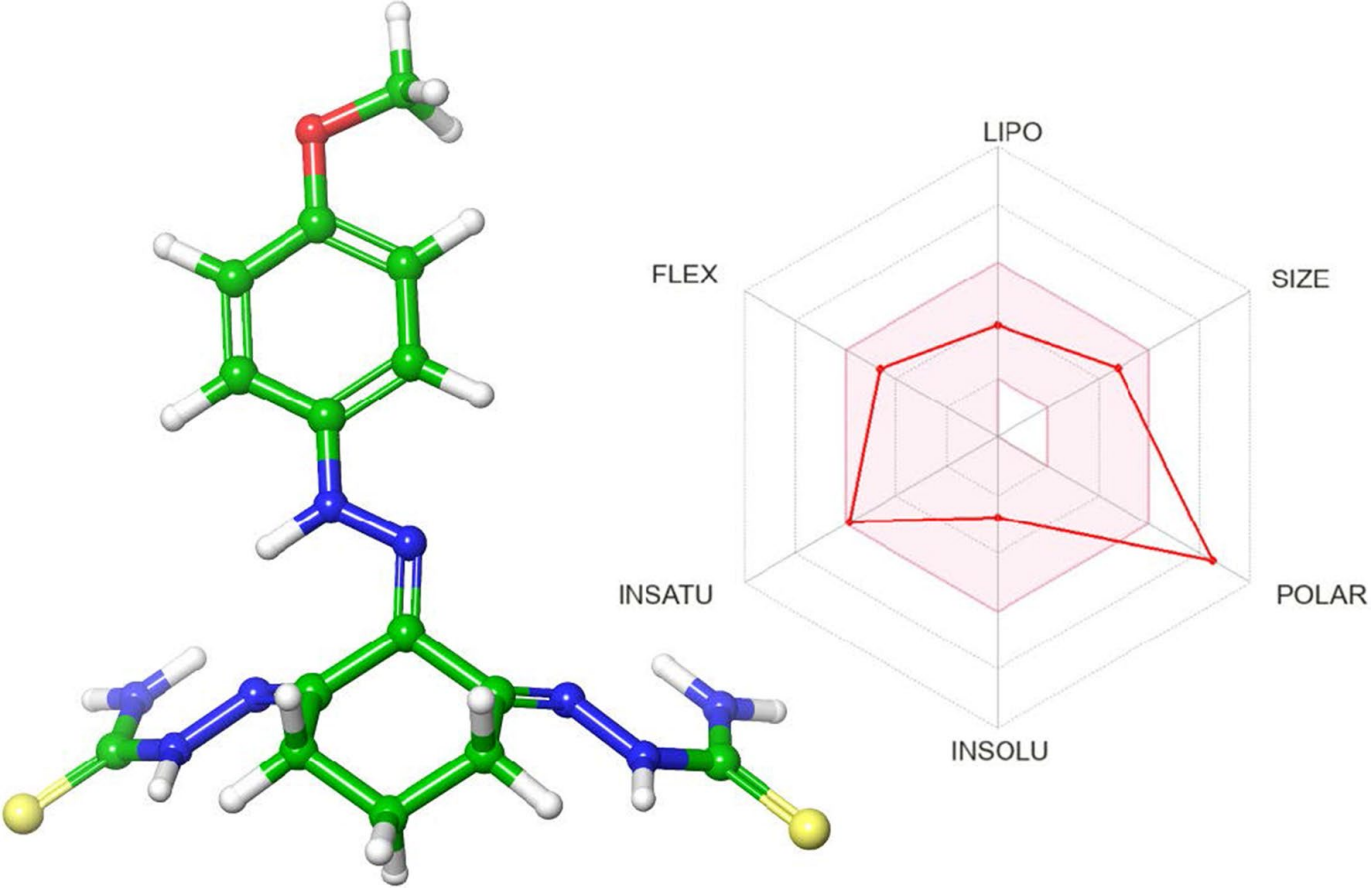
Color regions and pharmacological parameters of the **compound**. FLEX (Flexibility): 0 < Num. Rotatable bonds < 9, INSATU (Insaturation): 0.25 < Fraction Csp3 < 1, INSOLU (Indissolubility): 0 < LogS(ESOL) < 6, POLAR (Polarity): 20 Å^2^ < TPS < 130 Å^2^, LIPO (lipophilicity): − 0.7 < XLOG P3 + 5.0

Determining the pharmacokinetic properties of the molecule designed as an enzyme inhibitor and profiling the absorption, distribution, metabolism, and excretion (ADME) is an important preliminary study because of the success of the insertion experiments. In clinical trials, ADME properties of the active site(s) of a candidate drug should be in an appropriate ratio [65, 66]. Lipinski’s five rules were used to evaluate the efficacy of our phthalonitrile compound as a candidate drug [67–69], and the results are presented in Table 3. The rotatable bonds within the active groups of a drug candidate molecule increase flexibility and make it better adapted to the binding site. This moiety contains 5 swivel bonds and is sufficiently flexible. Log P, a measure of molecular hydrophobicity or lipophilicity, was around 5, which is considered reasonable for excellent permeability across the cell membrane. The overall properties of the compound are within Lipinski’s five rules of thumb for the number of hydrogen bond acceptors and donors [17–19, 70]. The color zone is the physicochemical area suitable for oral bioavailability in Fig. 12.

**Table 3:** Physicochemical and lipophilicity of the most active compound using SwissADME software

Code	Lipophilicity consensus log *P*	Physicochemical properties								
		MW (g/mol)	Heavy atoms	Aromatic heavy atoms	Rot. bond	H‐bond acc	H‐bond don	MR	TPSA (A^2^)	% ABS
**1a**	0.69	392.50	26	6	7	4	5	111.43	198.62	40.47

*MW* molecular weight, *MR* molar refractivity, *TPSA* topological polar surface area, *%ABS* percentage of absorption (%ABS = 109 − [0.345 × TPSA]

**Fig. 13:**
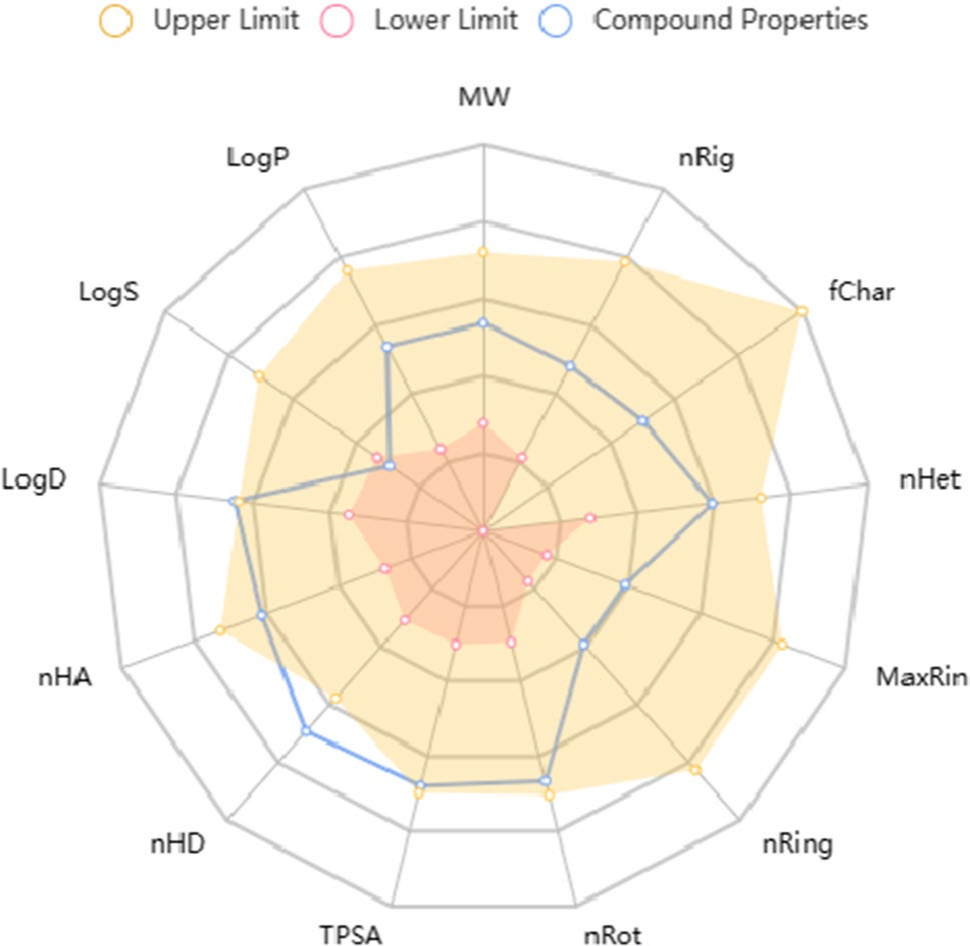
Drug-like rule prediction using the web application ADMETlab 2.0

Moreover, the proposed compound’s toxicity was evaluated using the ADMETlab 2.0 web program (Fig. 13). It has a great deal of potential to help medicinal chemists create new medications more quickly and prediction models for evaluating physicochemical properties and medicinal chemistry. The method that has drawn the most interest seeks to predict drug action by choosing suitable descriptors that roughly represent structure–activity relationships. The expected assessment of toxicophoric standards was used to determine proper and improper outcomes of the ligand in drug-like experiments. For example, certain effects have not been mitigated, such as acute toxicity, non-biodegradable, ocular corrosion, and eye discomfort. It has been discovered, although, that it is incompatible under specific circumstances. This method seeks to predict pharmacological activity by using suitable descriptors that illustrate structure–activity relationships [71].

## Conclusion

In this study; firstly, bis(thiosemicarbazone) ligand (L) was synthesized by the condensation reaction of thiosemicarbazide and a hydrazone derivative compound. Then, the novel Cu(II), Ni(II), Co(II), and Mn(II) complexes were synthesized by the reaction of obtained ligand (L). The structures of synthesized ligand and their complexes were characterized using elemental analysis, IR, UV–Vis, ^1^H-NMR spectra, ^13^C-NMR spectra, LC–MS, TGA-DTA, and differential scanning calorimetry techniques. Except L–Co(II) complex, the other complexes were binuclear. It was observed that all molecules were effective inhibitors for both enzymes. It was found that the complex molecules were better inhibitors than the standard inhibitors tacrine and EA. In addition, the ligand’s docking performance, interactions with AChE and GST enzyme receptors, and inhibition studies showed a significant correlation. In vitro and in silico studies have demonstrated that this compound holds potential for consideration in drug design processes.
